# Clinical Characteristics and Outcomes of Traumatic Brain Injury in a High-Volume Tertiary Care Center in India: A Prospective Observational Cohort Study

**DOI:** 10.1227/neu.0000000000003380

**Published:** 2025-02-21

**Authors:** Moritz Steinruecke, Shalini Nair, Sara Venturini, Fotios Siannis, Peter J. Hutchinson, Angelos Kolias, Mathew Joseph

**Affiliations:** * University of Cambridge School of Clinical Medicine, Cambridge, UK;; ‡ Department of Neurological Sciences, Christian Medical College Vellore, Vellore, Tamil Nadu, India;; § Division of Neurosurgery, Department of Clinical Neurosciences, University of Cambridge, Cambridge, UK;; ‖ NIHR Global Health Research Group on Acquired Brain and Spine Injury, University of Cambridge, Cambridge, UK;; ¶ Department of Mathematics, National and Kapodistrian University of Athens, Athens, Greece

**Keywords:** Global neurosurgery, India, Mortality, Outcome, Prognostication, Traumatic brain injury

## Abstract

**BACKGROUND AND OBJECTIVES:**

Traumatic brain injury (TBI) is a major public health challenge in India but there is a lack of high-quality data on its clinical characteristics and outcomes. We aimed to describe the TBI population of a tertiary care center in India, identify predictors of inpatient mortality, and assess the performance of existing prognostic tools.

**METHODS:**

We conducted a prospective observational cohort study of patients admitted to a high-volume tertiary care center in Vellore, India, after a TBI between 2013 and 2019.

**RESULTS:**

We identified 3172 patients (2667 males, 84%) admitted after a TBI (median age = 34 years [IQR 23-48]). Two-wheeler road traffic accidents caused 2259 (71%) injuries, in which 13 (0.6%) patients were wearing a helmet. There were 174 (5%) inpatient deaths (median length of stay = 6 days [IQR 4-10]) and overall mortality (median follow-up = 6 months [IQR 3-9]) was 17% (n = 540). Age, Glasgow Coma Scale motor score, systolic blood pressure ≤90 mm Hg, and key computed tomography imaging features were independently associated with inpatient mortality. Existing prognostic models predicted inpatient mortality with good performance (International Mission for Prognosis and Analysis of Clinical Trials in TBI: Brier = 0.0876, area under the curve (AUC) = 83% [95% CI 79%-87%]; Rotterdam CT: Brier = 0.0890, AUC 79% [95% CI 75%-83%]), but showed poorer performance for post-discharge mortality (International Mission for Prognosis and Analysis of Clinical Trials in TBI: Brier = 0.134, AUC = 75% [95% CI 72%-78%]; Rotterdam CT: Brier = 0.145, AUC 66% [95% CI 63%-69%]).

**CONCLUSION:**

In a tertiary care center in India, we described a predominantly young male TBI population with a high contribution of 2-wheeler road traffic accidents and significant post-discharge mortality. Existing prognostic models showed poor performance when predicting which patients died after discharge. These findings should inform public health interventions to reduce the significant burden of TBI in India.

**ABBREVIATIONS:**
CMC: Christian Medical College
ED: emergency department
GOS-E: GOS-Extended
HRs: hazard ratios
IMPACT: International Mission for Prognosis and Analysis of Clinical Trials in TBI
LMICs: low- and middle-income countries
TBI: traumatic brain injury.

Traumatic brain injury (TBI) is the most common neurosurgical emergency globally and a growing public health challenge in low- and middle-income countries (LMICs).^1,2^ There is a lack of high-quality TBI data in India, and incidence and mortality estimates vary widely.^3-6^ In 2016, the Global Burden of Disease Study estimated that over 3.5 million cases of TBI occur in India per year, which are associated with over 900 000 years of life lived with disability.^1^ These measures increased by 6% and 19% since 1990, respectively. Importantly, there is considerable variation in the characteristics of TBI across country income groups.^7^ In addition, many prognostic models have been developed in high-income countries and need to be validated in LMICs.^8,9^

A large proportion of TBI cases in LMICs can be prevented or made less severe through public health policies, particularly those addressing road safety and access to prehospital care. In India, in 2019, road injuries were the second leading cause of death among men between the ages of 15 and 49 years, and the fourth most common cause of death in this age group overall.^10^ TBI also causes significant long-term mortality and functional impairment, which has major socioeconomic implications.^11,12^

Granular data on the causes, management, and outcomes of TBI are required to inform public health policies and clinical trials in India. Here, we conducted a prospective observational cohort study to describe the clinical characteristics and medium-term functional outcomes of patients admitted to a high-volume tertiary care center in Vellore, India, after a TBI. In addition, we identified predictors of inpatient mortality and assessed the performance of prognostic models in moderate and severe TBI.

## METHODS

We conducted a prospective observational cohort study of all patients, including children, admitted to Christian Medical College (CMC) Vellore between March 28th, 2013, and September 29th, 2019, within 72 hours of a TBI, including those transferred from referring hospitals. We did not include patients who were admitted for observation in the emergency department (ED). Patients with severe abdominal or spine injuries were admitted to trauma and spine services, respectively, and were not included in the database. CMC Vellore is associated with a private, community-run tertiary care hospital located in Tamil Nadu, India, which admits patients with TBI within a radius of up to 250 km. We collected data on patient demographics, injury characteristics, acute clinical assessments, computed tomography (CT) features, and patient outcomes, measured using the Glasgow Outcome Scale-Extended (GOS-E), using a secure prospective database (**Supplemental Digital Content 1** [http://links.lww.com/NEU/E653]).^13^ Clinical assessments, including the Glasgow Coma Scale (GCS), were performed by the admitting clinician. CT scans were described based on the features included in the Rotterdam CT model, namely basal cistern appearance (normal, compressed, or absent), midline shift (≤5 mm or > 5 mm), an epidural mass lesion (present or absent), and intraventricular blood or traumatic subarachnoid hemorrhage (absent or present). Patients who were transferred from referring hospitals had their clinical and radiological assessments recorded on admission to CMC Vellore. GOS-E was assessed by a consultant neurosurgeon (MJ) upon discharge from routine follow-up.

TBI severity was classified based on patients' GCS on admission (mild = GCS of 13-15; moderate = GCS of 9-12; severe = GCS of <9). All patients with severe head injuries who did not undergo immediate surgery had intracranial pressure (ICP) monitoring via an extraventricular drain. Patients who did not receive an ICP monitor underwent more frequent imaging. Twenty percent mannitol was initially used for osmotherapy before 3% saline became the primary agent from 2017 onward. We aimed to discharge patients from routine outpatient follow-up if they had a GOS-E of 1 or 8 at any time; if they had a GOS-E of 2 at ≥1 month after discharge from hospital; or if they had a GOS-E of 3-7 at ≥6 months after discharge from hospital. An unfavorable outcome was defined as a GOS-E of ≤4 (upper severe disability or worse) at discharge from routine follow-up.

We compared the characteristics of patients who underwent craniotomy and decompressive craniectomy, and of patients who died as inpatients and after discharge. Patients who died after discharge did not have their date of death recorded in our database. Therefore, our survival analyses focused on inpatient mortality. We used log-rank tests and Cox proportional hazards models for survival analyses. Variables were chosen for the Cox proportional hazards model based on their clinical relevance and significance (*P* < .1) on univariate analysis. Patient sex was included in the model irrespective of significance on univariate analysis. We used backward elimination to remove nonsignificant (*P* > .1) variables from the model but retained nonsignificant variables that provided additional clinical context (eg, GCS components and CT imaging features). In our Cox proportional hazards model, GCS components were converted into continuous variables.

We evaluated the International Mission for Prognosis and Analysis of Clinical Trials in TBI (IMPACT) (core) and Rotterdam CT models for predicting overall, inpatient, and post-discharge mortality and unfavorable outcomes in patients with moderate or severe TBI. The IMPACT (core) model uses age, GCS motor score, and a pupils score to predict 6-month mortality or unfavorable outcomes in patients (≥14 years) with moderate or severe TBI (GCS on admission ≤12). The Rotterdam CT model uses CT imaging features (basal cistern appearance, midline shift, absence of an epidural mass lesion, and intraventricular blood or traumatic subarachnoid hemorrhage) on admission to predict 6-month mortality in patients (≥16 years) with moderate or severe TBI (GCS on admission ≤12). We used Brier scores for overall performance, the receiver operating characteristic (ROC) area under the curve (AUC) for discrimination, and Hosmer–Lemeshow tests for calibration.^8,9^ We did not use Hosmer–Lemeshow tests for the Rotterdam CT model because it classifies patients into only 6 mortality categories.

The registry for this study was approved by the Institutional Review Board at CMC Vellore. Consent from individual patients was not required because of the nature of the study. Statistical analysis was conducted using R (Version 4.1.1; R Core Team, 2021) and RStudio (Version 2022.7.0.548; RStudio Team, 2022). We used the survival() and survivalAnalysis() packages to conduct survival analyses.^14,15^ We described nonparametric continuous data using medians and IQRs. We described our Cox proportional hazards model using hazard ratios (HRs), 95% CIs, and *P* values. We specified where data were missing from analyses. We set an α level of 0.05 (2-sided) for statistical significance and used the Bonferroni correction for multiple comparisons. We reported our findings using Strengthening the Reporting of Observational Studies in Epidemiology guidelines.

## RESULTS

Between March 28th, 2013, and September 29th, 2019, 3172 patients (2667 males, 84%) were admitted after a TBI (Table 1). Patients had a median age of 34 years (IQR 23-48) and a median of 10 years of education (IQR 6-13, n = 3076). Two-wheeler road traffic accidents, including those involving motorcycles and seated scooters, were the most common cause of injury (n = 2259, 71%). Patients were wearing a helmet in 13 (0.6%) of these cases and reported alcohol consumption in 714 (32%) cases. Less common mechanisms of injury included falls (n = 307, 10%), pedestrian–vehicle accidents (n = 278, 9%), and 4-wheeler road traffic accidents (n = 163, 5%). The median time from injury to arrival at the ED was 3 hours (IQR 2-7). One thousand ninety-three (34%) patients were transferred from a referring hospital. Based on their GCS on admission, 1162 (37%) patients experienced a mild injury (GCS = 13-15), 1042 (33%) a moderate injury (GCS = 9-12), and 968 (31%) a severe injury (GCS <9).

**TABLE 1.** Population Characteristics by TBI Severity

	All patients (n = 3172)	TBI severity		
		Mild (n = 1162, 37%)	Moderate (n = 1042, 33%)	Severe (n = 968, 31%)
Age (y)	34 (IQR 23-48)	35 (IQR 24-49)	34 (IQR 23-48)	31 (IQR 22-47)
Sex (male)	2667 (84%)	943 (81%)	867 (83%)	858 (89%)
Mode of injury				
Two-wheeler	2259 (71%)	788 (68%)	761 (73%)	710 (73%)
Four-wheeler	163 (5%)	51 (4%)	54 (5%)	58 (6%)
Vehicle–pedestrian	278 (9%)	98 (8%)	93 (9%)	87 (9%)
Fall	307 (10%)	150 (13%)	88 (8%)	69 (7%)
Assault	53 (2%)	31 (3%)	18 (2%)	4 (0.4%)
Other	112 (4%)	44 (4%)	28 (3%)	40 (4%)
Time to ED (h)	3 (IQR 2-7)	4 (IQR 2-9)	3 (IQR 2-7)	3 (IQR 2-5)
Airway not maintained or intubated on arrival	804 (25%)	4 (0.3%)	107 (10%)	693 (72%)
Pupil(s) nonreactive or unequal on admission	758 (24%)	131 (11%)	183 (18%)	444 (46%)
GCS on admission	11 (IQR 8-14)	14 (IQR 13-15)	10 (IQR 9-11)	7 (IQR 4-8)
Systolic blood pressure ≤90 mm Hg on admission	220 (7%)	46 (4%)	58 (6%)	116 (12%)
Cistern appearance				
Compressed	838 (26%)	260 (22%)	317 (30%)	261 (27%)
Absent	291 (9%)	18 (2%)	60 (6%)	213 (22%)
Midline shift > 5 mm	480 (15%)	111 (10%)	126 (12%)	243 (25%)
Epidural mass	256 (8%)	122 (10%)	74 (7%)	60 (6%)
Intraventricular blood or tSAH	1204 (28%)	304 (26%)	406 (39%)	494 (51%)
Operation				
None	2363 (74%)	898 (77%)	762 (73%)	703 (73%)
Craniotomy	222 (7%)	94 (8%)	74 (7%)	54 (6%)
DC	16 (0.5%)	3 (0.3%)	4 (0.4%)	9 (0.9%)
DC + evacuation	405 (13%)	85 (7%)	158 (15%)	162 (17%)
Depressed fracture	116 (4%)	68 (6%)	27 (3%)	21 (2%)
Other	50 (2%)	14 (1%)	17 (2%)	19 (2%)
ICP monitoring	159 (5%)	2 (0.2%)	26 (2%)	131 (14%)
Tracheostomy	500 (16%)	40 (3%)	116 (11%)	344 (36%)
Ventilation	1314 (41%)	157 (14%)	350 (34%)	807 (83%)
Length of stay (d)	6 (IQR 4-10)	5 (IQR 3-8)	7 (IQR 4-11)	7 (IQR 3-11)
GCS at discharge	15 (IQR 12-15)	15 (IQR 15-15)	14 (IQR 13-15)	11 (IQR 4-14)
Adm–dis GCS change	+2 (IQR 0-4)	0 (IQR 0-1)	+4 (IQR 3-5)	+4 (IQR 0-7)
IMPACT (core) expected 6-month mortality (n = 1796)	—	—	152 (16%)	272 (32%)
Rotterdam CT expected 6-month mortality (n = 1787)	—	—	159 (17%)	222 (26%)
Inpatient mortality	174 (5%)	15 (1%)	27 (3%)	132 (14%)
Overall mortality	540 (17%)	65 (6%)	111 (11%)	364 (39%)
GOS-E at discharge from routine follow-up (n = 3082)	8 (IQR 5-8)	8 (IQR 8-8)	8 (IQR 6-8)	5 (IQR 1-8)
Good recovery (GOS-E = 7-8) (n = 3082)	2066 (67%)	954 (84%)	736 (73%)	376 (40%)

CT, computed tomography; DC, decompressive craniectomy; ED, emergency department; GCS, Glasgow Coma Scale; GOS-E, Glasgow Outcome Scale-Extended; ICP, intracranial pressure; IMPACT, International Mission for Prognosis and Analysis of Clinical Trials in TBI; TBI, traumatic brain injury; tSAH, traumatic subarachnoid hemorrhage.

Cistern appearance, midline shift, epidural mass, and intraventricular blood or tSAH refer to features on the initial CT scan. Mild TBI: GCS = 13-15 on admission; moderate TBI: GCS = 9-12 on admission; severe TBI: GCS <9 on admission. Continuous data are presented as median (IQR).

The median time from injury to first CT scan was 4 hours (IQR 3-6). The most common findings on initial imaging were presence of intraventricular or traumatic subarachnoid hemorrhage (n = 1204, 28%), compressed basal cisterns (n = 838, 26%), and midline shift > 5 mm (n = 480, 15%). Basal cisterns were absent in 291 (9%) patients and epidural mass lesions were present in 256 (8%) patients.

Eight hundred nine (26%) patients underwent surgery. The most common operations were decompressive craniectomy ± hematoma evacuation (n = 421, 52%), in which the bone flap is removed, craniotomy and hematoma evacuation (n = 222, 27%), in which the bone flap is replaced, and elevation and debridement of a depressed skull fracture (n = 116, 14%). Demographics, TBI severity, acute clinical assessments, CT findings, and outcomes varied between patients who underwent craniotomy and decompressive craniectomy (Table 2).

**TABLE 2.** Characteristics of Patients Who Underwent Craniotomy or Decompressive Craniectomy

	Craniotomy (n = 222)	Decompressive craniectomy (n = 421)	Comparison between groups (*P* value)
Age (y)	29 (IQR 20-42)	37 (IQR 27-48)	<0.001^a^
Sex (male)	203 (91%)	331 (79%)	<0.001^a^
TBI severity			
Mild	94 (42%)	88 (21%)	<0.001^a^
Moderate	74 (33%)	162 (38%)	
Severe	54 (24%)	171 (41%)	
Airway not maintained or intubated on arrival	42 (19%)	151 (36%)	<0.001^a^
Pupil(s) nonreactive or unequal on admission	46 (21%)	115 (27%)	0.0665
GCS on admission	11 (IQR 9-14)	9 (IQR 8-11)	<0.001^a^
Systolic blood pressure ≤90 mm Hg on admission	17 (8%)	13 (3%)	0.0133
Cistern appearance			
Compressed	125 (56%)	258 (61%)	<0.001^a^
Absent	27 (12%)	117 (28%)	
Midline shift >5 mm	102 (46%)	229 (54%)	0.0416
Epidural mass	144 (65%)	30 (7%)	<0.001^a^
Intraventricular blood or tSAH	61 (27%)	244 (58%)	<0.001^a^
Length of stay (d)	5 (IQR 3-10)	10 (IQR 7-15)	<0.001^a^
ICP monitoring	6 (3%)	27 (6%)	0.0560
Tracheostomy	20 (9%)	183 (43%)	<0.001^a^
Ventilation	113 (51%)	416 (99%)	<0.001^a^
GCS at discharge	15 (IQR 14-15)	12 (IQR 8-14)	<0.001^a^
Adm–dis GCS change	+2 (IQR 0-5)	+2 (IQR 0-4)	<0.001^a^
Inpatient mortality	5 (2%)	30 (7%)	0.00960
Overall mortality	16 (7%)	135 (32%)	<0.001^a^
GOS-E at discharge from routine follow-up (n = 3082)	8 (IQR 7-8)	5 (IQR 1-8)	<0.001^a^
Good recovery (GOS-E = 7-8) (n = 3082)	172 (79%)	160 (39%)	<0.001^a^

CT, computed tomography; GCS, Glasgow Coma Scale; GOS-E, Glasgow Outcome Scale-Extended; ICP, intracranial pressure; TBI, traumatic brain injury; tSAH, traumatic subarachnoid hemorrhage.

a Significance at a Bonferroni-corrected α level of 0.002. χ^2^ tests were used to compare categorical variables between groups and t-tests to compare means between groups.

Cistern appearance, midline shift, epidural mass, and intraventricular blood or tSAH refer to features on the initial CT scan. Mild TBI: GCS = 13-15 on admission; moderate TBI: GCS = 9-12 on admission; severe TBI: GCS <9 on admission. Continuous data are presented as median (IQR).

**FIGURE 1.**
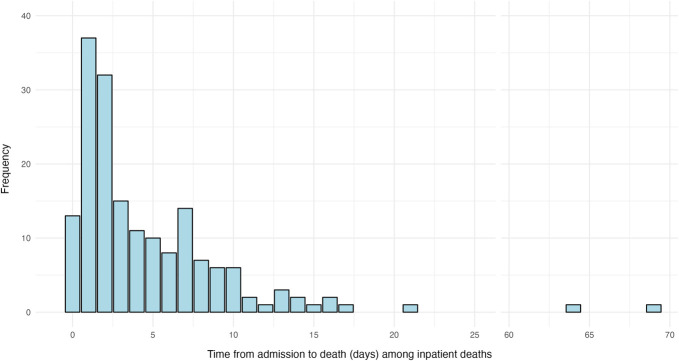
Time from admission to death among patients who died in hospital (n = 174).

**FIGURE 2.**
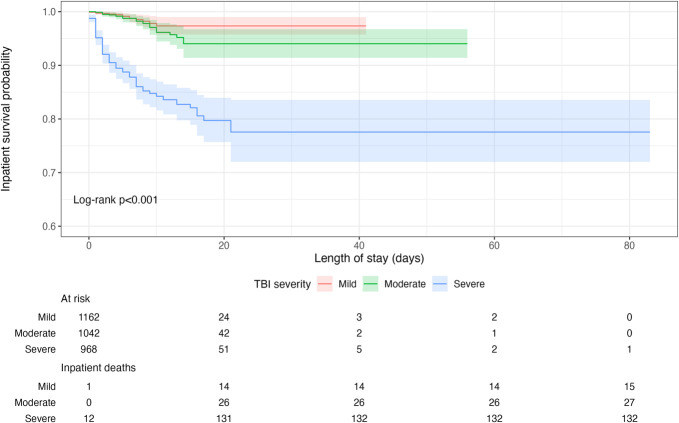
Kaplan–Meier curves displaying inpatient survival by TBI severity. Mild TBI: GCS = 13-15 on admission; moderate TBI: GCS = 9-12 on admission; severe TBI: GCS <9 on admission. GCS, Glasgow Coma Scale; TBI, traumatic brain injury.

The median length of stay was 6 days (IQR 4-10). The median change in GCS from admission to discharge was +2 points (IQR 0-4). One hundred seventy-four (5%) patients died during the index admission. Among inpatient mortalities, 82 (47%) patients died within 2 days of admission, 108 (62%) within 4 days, and 147 (84%) within 8 days (Figure 1). There was a second peak in mortality at 7 days after admission (n = 14). Inpatient survival differed in relation to TBI severity (*P* < .001) (Figure 2). One hundred fifteen (4%) patients had health insurance, and 994 (31%) patients were unable to cover their hospital expenses. The median hospital expenditure was 35 850 Indian Rupees (IQR 17000-95625) and the median patient expenditure was 28 900 Indian Rupees (IQR 14700-68000).

**FIGURE 3.**
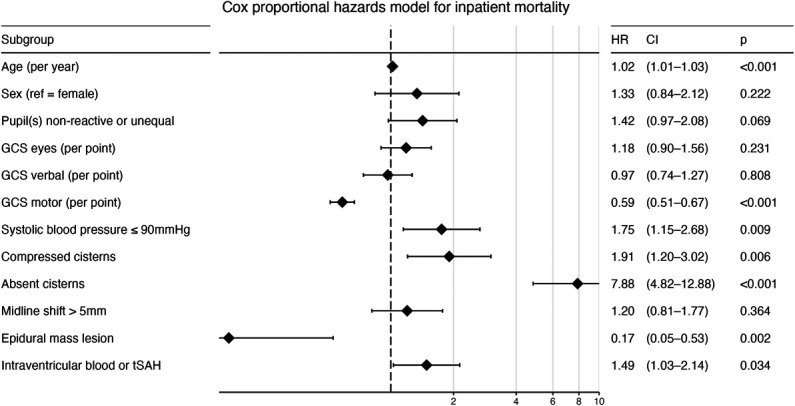
Cox proportional hazards model for inpatient mortality (3172 patients, 174 inpatient deaths). Reference categories for dichotomous variables: sex = female; pupil(s) nonreactive or unequal = pupils reactive and equal; systolic blood pressure ≤90 mm Hg = systolic blood pressure ≥90 mm Hg; compressed and absent basal cisterns = normal; midline shift >5 mm = midline shift ≤5 mm; epidural mass lesion = no epidural mass lesion; intraventricular blood or traumatic subarachnoid hemorrhage = no intraventricular hemorrhage and traumatic subarachnoid hemorrhage. CI, 95% confidence interval; GCS, Glasgow Coma Scale; HR, hazard ratio for inpatient mortality; tSAH, traumatic subarachnoid hemorrhage.

A Cox proportional hazards model of 3172 patients (174 inpatient deaths) associated age (HR 1.02 per year, 95% CI 1.01-1.03), GCS motor score (HR 0.59 per point, 95% CI 0.51-0.67), systolic blood pressure ≤90 mm Hg (HR 1.75, 95% CI 1.15-2.68), basal cistern appearance (compressed: HR 1.91, 95% CI 1.20-3.02; absent: HR 7.88, 95% CI 4.82-12.88), an epidural mass lesion (HR 0.17, 95% CI 0.05-0.53), and intraventricular blood or traumatic subarachnoid hemorrhage (HR 1.49, 95% CI 1.03-2.14) with inpatient mortality (Figure 3, **Supplemental Digital Content 2** [http://links.lww.com/NEU/E654]).

**FIGURE 4.**
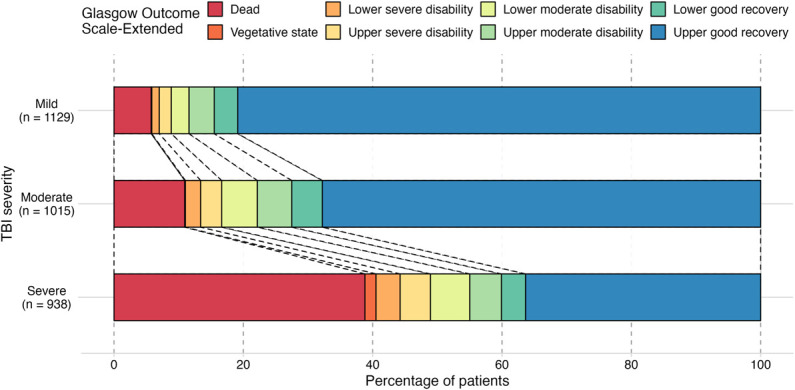
Glasgow Outcome Scale-Extended at discharge from routine follow-up. Mild TBI: GCS = 13-15 on admission; moderate TBI: GCS = 9-12 on admission; severe TBI: GCS <9 on admission. GCS, Glasgow Coma Scale; TBI, traumatic brain injury.

Overall, 2066 (67%) patients with a recorded GOS-E (n = 3082) had a good recovery (GOS-E = 7-8). Among patients who survived to hospital discharge (n = 2998), 2541 (85%) had a GOS-E and follow-up time recorded. Among these patients, the median outpatient follow-up period was 6 months (IQR 3-9). An additional 366 (12%) patients died during the routine follow-up period. Overall mortality was 17% (n = 540). Twenty-nine (1%) patients received formal rehabilitative care after discharge from hospital. TBI severity (mild, moderate, or severe) was associated with GOS-E at discharge from routine follow-up (Figure 4). In comparison with patients who died as inpatients, those who died after discharge from hospital had a higher average GCS on admission, less severe CT findings, and were more likely to receive a tracheostomy (Table 3).

**TABLE 3.** Population Characteristics of Patients Who Died in Hospital and After Discharge

	Inpatient mortality (n = 174)	Post-discharge mortality (n = 366)	Comparison between groups (*P* value)
Age (y)	42 (IQR 27-57)	45 (IQR 28-58)	0.546
Sex (male)	152 (87%)	310 (85%)	0.693
Mode of injury			
Two-wheeler	120 (69%)	245 (67%)	0.361
Four-wheeler	12 (7%)	21 (6%)	
Vehicle–pedestrian	21 (12%)	44 (12%)	
Fall	10 (6%)	41 (11%)	
Assault	3 (2%)	3 (0.8%)	
Other	8 (5%)	12 (3%)	
TBI severity			
Mild	15 (9%)	50 (14%)	0.0151
Moderate	27 (16%)	84 (23%)	
Severe	132 (76%)	232 (63%)	
Airway not maintained or intubated on arrival	124 (71%)	221 (60%)	0.0139
Pupil(s) nonreactive or unequal on admission	107 (61%)	176 (48%)	0.00355
GCS on admission	4 (IQR 3-8)	8 (IQR 4-10)	<0.001^a^
Systolic blood pressure ≤90 mm Hg on admission	28 (16%)	34 (9%)	0.0205
Cistern appearance			
Compressed	42 (24%)	117 (32%)	<0.001^a^
Absent	91 (52%)	87 (24%)	
Midline shift > 5 mm	84 (48%)	103 (28%)	<0.001^a^
Epidural mass	3 (2%)	15 (4%)	0.151
Intraventricular blood or tSAH	126 (72%)	224 (61%)	0.109
Operation			
None	136 (78%)	242 (66%)	0.103
Craniotomy	5 (3%)	11 (3%)	
DC	1 (0.6%)	2 (0.5%)	
DC + evacuation	29 (17%)	103 (28%)	
Depressed fracture	2 (1%)	4 (1%)	
Other	1 (0.6%)	4 (1%)	
ICP monitoring	10 (6%)	50 (14%)	0.00624
Tracheostomy	42 (24%)	157 (43%)	<0.001^a^
Ventilation	162 (94%)	316 (86%)	0.0212
Length of stay (d)	3 (IQR 1-7)	6 (IQR 2-10)	0.00429
GCS at discharge	—	6 (IQR 3-10)	—
Adm–dis GCS change	—	0 (IQR -3-2)	—
IMPACT (core) Brier score (n = 1796)	0.0876	0.134	—
Rotterdam CT Brier score (n = 1787)	0.0890	0.145	—
IMPACT (core) AUC (n = 1796)	83.1% (95% CI 79.2%-86.9%)	75.2% (95% CI 72.3%-78.1%)	—
Rotterdam CT AUC (n = 1787)	79.3% (95% CI 75.1%-83.4%)	65.8% (95% CI 62.5%-69.0%)	—

CT, computed tomography; ED, emergency department; GCS, Glasgow Coma Scale; tSAH, traumatic subarachnoid hemorrhage; DC, decompressive craniectomy; ICP, intracranial pressure; AUC, area under the curve; IMPACT, International Mission for Prognosis and Analysis of Clinical Trials in TBI; TBI, traumatic brain injury.

a Significance at a Bonferroni-corrected α level of 0.0029. χ^2^ tests were used to compare categorical variables between groups and *t*-tests to compare means between groups.

Cistern appearance, midline shift, epidural mass, and intraventricular blood or tSAH refer to features on the initial CT scan. Mild TBI: GCS = 13-15 on admission; moderate TBI: GCS = 9-12 on admission; severe TBI: GCS <9 on admission. Data presented as median (IQR).

Next, we evaluated the IMPACT (core) and Rotterdam CT prognostic models for predicting mortality and unfavorable outcomes (GOS-E 1-4) in our data set. We had 1796 patients who met criteria for the IMPACT (core) model and 1787 patients who met criteria for the Rotterdam CT model.

We used Brier scores to assess overall model performance. The IMPACT (core) model had the following Brier scores: overall mortality = 0.133; inpatient mortality = 0.0876; post-discharge mortality = 0.134; unfavorable outcomes = 0.177. We also calculated Brier scores for the Rotterdam CT model (overall mortality = 0.159; inpatient mortality = 0.0890; post-discharge mortality = 0.145).

**FIGURE 5.**
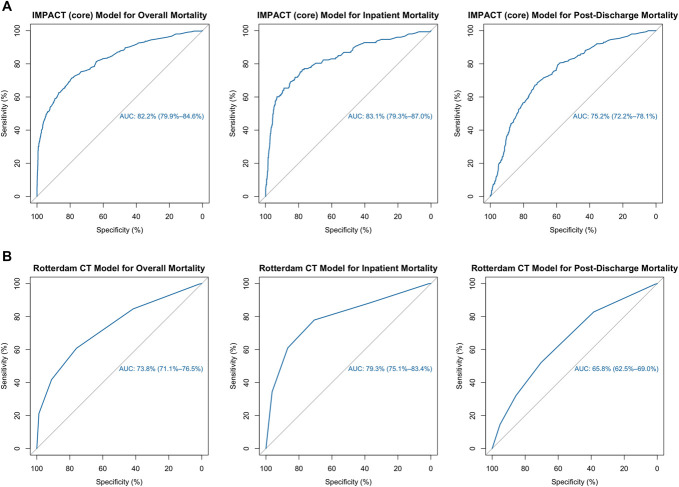
Evaluation of existing prognostic models for predicting overall, inpatient, and post-discharge mortality. **A**, IMPACT (core) model: patients ≥14 years with moderate or severe TBI (GCS on admission ≤12) (n = 1796). **B**, Rotterdam CT model: patients ≥16 years with moderate or severe TBI (GCS on admission ≤12) (n = 1787). 95% confidence interval presented in brackets. AUC, area under the curve; CT, computed tomography; GCS, Glasgow Coma Scale; IMPACT, International Mission for Prognosis and Analysis of Clinical Trials in TBI; TBI, traumatic brain injury.

We used the ROC AUC to assess model discrimination. The ROC AUC for the IMPACT (core) model was 82.2% (95% CI 79.9%-84.6%) for overall mortality, 83.1% (95% CI 79.3%-87.0%) for inpatient mortality, 75.2% (95% CI 72.2%-78.1%) for post-discharge mortality, and 81.1% (95% CI 78.9%-83.4%) for unfavorable outcomes (Figure 5A). The ROC AUC for the Rotterdam CT model was 73.8% (95% CI 71.1%-76.5%) for overall mortality, 79.3% (95% CI 75.1%-83.4%) for inpatient mortality, and 65.8% (95% CI 62.5%-69.0%) for post-discharge mortality (Figure 5B).

Finally, we assessed calibration of the IMPACT (core) model using Hosmer–Lemeshow tests (overall mortality statistic = 7.84, *P* = .449; inpatient mortality statistic = 7.23, *P* = .513; post-discharge mortality statistic = 49.5, *P* < .001; unfavorable outcome statistic = 6.32, *P* = .611).

## DISCUSSION

In a large tertiary care center in India, we identified a predominantly young male TBI population with a high contribution of 2-wheeler road traffic accidents. Inpatient mortality was 5%, but approximately a quarter of patients died or experienced unfavorable outcomes by the end of the median 6-month follow-up period. Advanced age, GCS motor score, systolic shock on admission, and key CT imaging features were associated with inpatient mortality. Compared with inpatient mortality, existing prognostic models showed poorer performance when predicting which patients died after discharge.

Two systematic reviews of TBI studies in India identified predominantly young male cohorts with a large contribution of road traffic accidents.^5,6^ However, both reviews identified significant variation in data-reporting standards, and few large studies described the characteristics of all patients admitted to hospital after a TBI, which makes it challenging to directly compare cohorts. Our granular clinical data, identification of predictors of inpatient mortality, and evaluation of prognostic models add to the literature in this field.

The TBI populations of tertiary care centers in India have changed over the past 30 years. The proportion of cases caused by 2-wheeler road traffic accidents has increased since the 1990s, likely because of significant motorization during this period.^16^ The average severity of TBI cases seems to have increased substantially, although we did not include patients with mild injuries who were admitted for observation in the ED. Nevertheless, outcomes have likely improved, with lower inpatient mortality and more patients achieving a good recovery in our cohort than in a report from Bangalore in 1994.^17^

The characteristics of our TBI population also differ from those seen in high-income countries, where patients are generally older, incidental falls are a more common etiology, and injuries are on average less severe.^18^ In the admission and intensive care unit strata of the CENTER-TBI core study, inpatient mortality was 10%, higher than we reported, but overall mortality was lower than in our cohort at 15%. A proportion of post-discharge deaths in our study can be attributed to our practice of informing patients' families of poor prognosis early in their care and their wishes to discontinue active treatment. However, there is also a need for improved access to rehabilitative services after discharge.

We also evaluated the IMPACT (core) and Rotterdam CT outcome prediction models using our data set. Both models were developed using data from high-income countries, so it is important to assess their performance in LMICs. Both models predicted overall and inpatient mortality with comparable performance to their initial reports in the literature.^8,9^ However, the models were less able to predict post-discharge mortality. Identifying factors that are associated with post-discharge mortality and unfavorable outcomes in LMICs will enable follow-up care to be tailored toward patients at high risk of deterioration.

Our study also describes the operative management of TBI in a tertiary care center in India. Decompressive craniectomy was preferred over craniotomy in patients with more severe presentations and had been performed more frequently in patients who experienced unfavorable outcomes. Randomized controlled trial evidence suggests that decompressive craniectomies and craniotomies are associated with comparable outcomes in patients with traumatic acute subdural hematoma, which led to a recommendation against preemptive decompressive craniectomies.^19^ A comparative effectiveness study using the CENTER-TBI and Net-QuRe cohorts found that centers preferring decompressive craniectomy over craniotomy did not have better outcomes and had higher reoperation and complication rates.^20^ However, these findings must be interpreted within the context of resource constraints in LMICs, such as limited access to ICP monitoring. In our cohort, just 5% of patients received ICP monitoring, which helps explain the tendency toward decompressive craniectomy.

This work identifies public health measures that could be effective at reducing the burden of TBI in India. Over 70% of all TBI cases in our cohort were attributable to 2-wheeler road traffic accidents in which patients were not wearing a helmet, and in approximately a third of these cases, patients reported alcohol consumption. The Motor Vehicles Act of 1988 mandates individuals riding 2-wheeler vehicles in India to wear a helmet, but there have been challenges with compliance.^21-23^ Academic, professional, and nongovernmental campaigns have aimed to promote helmet use on 2-wheeler vehicles, but their efficacy remains uncertain. In high-income countries, compliance with helmet laws has been shown to reduce the incidence and severity of TBI.^24-26^ Importantly, there is a lack of reliable data on both compliance with helmet laws and temporal trends in the incidence and severity of TBI in India. TBI registries, for example, by the National Institute of Mental Health and Neurosciences in Bangalore and Narayana Medical College and Hospital in Andhra Pradesh, have provided some insight, but further regional and national databases are required to effectively integrate information across health and social care services.^27-30^ Without routinely collecting hospital- and community-level data on the TBI population, it will be challenging to assess the effectiveness of public health and clinical interventions.

### Limitations

Our study has a few important limitations. First, we did not record a date of death for patients who died after leaving hospital. Therefore, our survival analyses focused primarily on inpatient mortality. Long-term mortality after TBI is challenging to predict, so these data would have been informative. Second, we did not collect structured data on patients' wider socioeconomic circumstances, which will have likely mediated their functional outcomes. Describing the socioeconomic burden of TBI in India is key for promoting effective public policies that require government investment.^31^ Finally, our study was conducted in a tertiary care center and did not include patients discharged from the ED, so is not representative of the TBI population across India.

## CONCLUSION

In a high-volume tertiary care center in India, we described a predominantly young male TBI population with a high contribution of 2-wheeler road traffic accidents and significant post-discharge mortality. We also identified clinical features associated with inpatient mortality and found that existing prognostic models performed poorly when predicting which patients died after leaving hospital. Further work is required to identify and implement effective public health interventions to reduce the significant health burden of TBI in India. In addition, the long-term disability associated with TBI in India warrants further study, and the provision of rehabilitative care after discharge from hospital must be increased.

## SUPPLEMENTAL DIGITAL CONTENT

**Supplemental Digital Content 1**. **Supplemental Methods**. Variables recorded in the database and their specification. **Supplemental Digital Content 2**. **Supplemental Figure**. Univariate log-rank tests used to select variables for modelling inpatient mortality.
